# The complete mitochondrial genome of *Glycera chirori* Izuka (Annelida: Polychaeta): an evidence of conservativeness between gene arrangement and phylogenesis in *Glycera*

**DOI:** 10.1080/23802359.2019.1681318

**Published:** 2019-10-24

**Authors:** Panpan Chen, Xin Shen, Yuefeng Cai, Nanjing Ji, Yongqi Li, Tian Ge, Shishi Liu

**Affiliations:** a Jiangsu Key Laboratory of Marine Bioresources and Environment/Jiangsu Key Laboratory of Marine Biotechnology, Jiangsu Ocean University, Lianyungang, China;; b Co-innovation Center of Jiangsu Marine Bio-industry Technology, Jiangsu Ocean University, Lianyungang, China

**Keywords:** *Glycera chirori* Izuka, mitochondrial genome, gene arrangement, phylogeny

## Abstract

The complete mitochondrial genome of *Glycera chirori* Izuka (Annelida: Polychaeta) was presented, which is a circular molecule of 15,930 bp nucleotides. It encodes 37 genes, including 13 PCGs, 22 tRNAs, and two rRNAs. The length of non-coding regions is 1428 bp, and the longest one (1346 bp) is speculated as the control region, which is located between *trnA* and *trnL_2_* and is longer than most species in *Glycera*. The complete mitogenome of *G*. *chirori* Izuka consists of 31.2% A, 23.6% C, 12.9% G, and 32.2% T, which has T vs. A skew (−0.02) and C vs. G skew (−0.29), respectively. Phylogenetic analysis indicates the classification status of *G. chirori* Izuka and the relationship with other species in *Glycera*, which is closer with *Glycera unicornis* and *Glycera fallax* (bootstrap = 100). By comparisons, the gene arrangement of *G. chirori* Izuka and other seven species in *Glycera* are identical and they also cluster together in phylogenetic tree with higher support rate, which indicates the conservativeness between gene arrangement and phylogenetic analysis in *Glycera*. In conclusion, the complete mitochondrial genome of *G. chirori* Izuka can provide supportive data for further molecular and evolutionary analysis of *Glycera*.

*Glycera* is one of three genera in Glyceridae and is also the most species-rich genus at present (Richter et al. 2015). There are some reports on this genus, which involved geographical distribution, biological activity, and molecular basis (Schüller 2011; von Reumont et al. 2014; Richter et al. 2015). *Glycera chirori* Izuka (Annelida: Polychaeta: Glyceridae) is one of the most common species in Glyceridae (Sun and Yang 1994), distributing in China's coastal water and Japan's sea area and living in the intertidal and subtidal zones (Wang and Song 2017).

The specimen of *G. chirori* Izuka was collected from intertidal of Multi-Island Sea, Lanshan District, Rizhao, Shandong Province, China (N: 30.71, E: 122.78). The total DNA was stored at Marine Museum of Jiangsu Ocean University (Accession number: Gchi-002). We used ultrasonic to interrupt long DNA fragments to 2–3 kb to build a library and get the complete mitochondrial genome sequence by sequencing and assembly with SeqMan 7.1.0 software (Swindell and Plasterer 1997). Gene annotation was obtained with MITOS (Bernt et al. 2013) and tRNAscan-SE (Chan and Lowe 2019).

The total length of mitogenome of *G. chirori* Izuka is 15,930 bp, which is a circular molecule, encoding 13 PCGs, two rRNA, and 22 tRNA genes on one strand (GenBank accession number: MK858188) (Boore 1999; Shen et al. 2009). AT and GC skews of the whole genome are −0.02 and −0.29, respectively (Perna and Kocher 1995). The length of all non-coding regions is 1428 bp. The Tandem Repeats Finder (Benson 1999) was used to speculate the control region (1346 bp), locating between *trnA* and *trnL_2_*, which is longer than most species in *Glycera* (Richter et al. 2015).

Gene arrangement was a useful tool to elucidate the evolution and phylogenetic relationship between homologous species (Boore 1999; Weigert et al. 2016). Compared with the other 12 species of *Glycera* in the GenBank database, the gene arrangement of *G. chirori* Izuka is identical in 13 PCGs and two rRNA genes and this arrangement pattern (15 genes) is consistent with most species in Annelida (Jennings and Halanych 2005; Zhong et al. 2008; Weigert et al. 2016). Besides, only a few tRNA genes show translocation for their higher variability (Weigert et al. 2016).

We used MEGA 7.0.25 (Kumar et al. 2016) to construct phylogenetic trees based on neighbour-joining (NJ) and maximum-likelihood (ML) methods, using 13 PCGs’s amino acid data from 13 species (*Glycera*) and four Oligochaeta species (outgroup) (Figure 1). As result showed, *G. chirori* Izuka clusters with *Glycera unicoins* isolated FS15 (BP = 92/95), and the two species cluster with *Glycera fallax* isolated FS14 (BP = 97/98). And then this group is clustered with the other two groups from other five species with high support rate (BP = 99). Significantly, *G. chirori* Izuka and other six species grouped on phylogenetic tree share the same gene arrangement, which indicated that gene arrangement and phylogenetic tree *in Glycera* complement each other. Thus, *G. chirori* Izuka could be a valid support to reflect the conservativeness between gene arrangement and phylogenetic analysis in *Glycera*.

**Figure 1.**
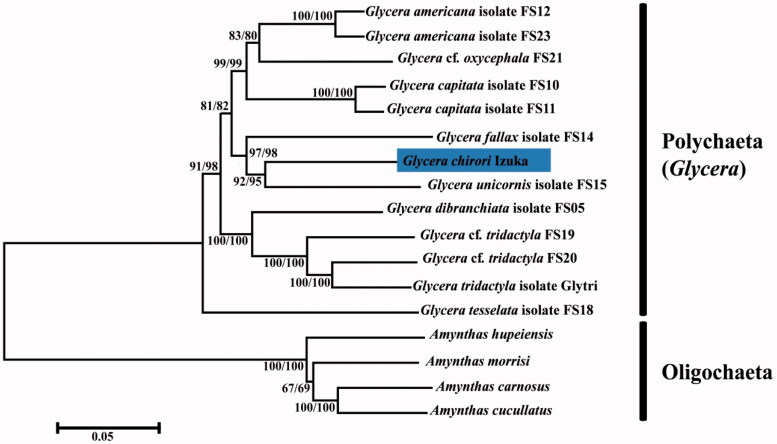
Phylogenetic trees constructed from neighbour-joining (NJ) and maximum-likelihood (ML) methods of 13 PCGs (amino acid data). The numerical values at the node represent the bootstrap value from ML and NJ methods, respectively.

The accession numbers of the genomes used for comparison were KT989321 (*G. americana* isolate FS12), KT989330 (*G. americana* isolate FS23), KT989329 (*G* cf. *oxycephola* FS21), KT989319 (*G. capitata* isolate FS10), KT989320 (*G. capitata* isolate FS11), KT989323 (*G. fallax* isolate FS14), KT989324 (*G. unicornis* isolate FS15), KT989318 (*G. dibranchiata* isolate FS15), KT989327 (*G* cf. *tridactyla* FS19), KT989328 (*G* cf. *tridactyla* FS20), KT989331 (*G*. *tridactyla* isolate Glytri), KT989326 (*G*. *tesselata* isolate FS18), NC_029864 (*Amynthas hupeiensis*), NC_029865 (*Amynthas morrisi*), NC_029863 (*Amynthas carnosus*), and NC_029866 (*Amynthas cucullatus*).
